# Revised Selection Criteria for Candidate Restriction Enzymes in Genome Walking

**DOI:** 10.1371/journal.pone.0035117

**Published:** 2012-04-11

**Authors:** Ali Taheri, Stephen J. Robinson, Isobel Parkin, Margaret Y. Gruber

**Affiliations:** Agriculture and Agri-Food Canada, Saskatoon Research Centre, Saskatoon, Saskatchewan, Canada; Pennsylvania State University, United States of America

## Abstract

A new method to improve the efficiency of flanking sequence identification by genome walking was developed based on an expanded, sequential list of criteria for selecting candidate enzymes, plus several other optimization steps. These criteria include: step (1) initially choosing the most appropriate restriction enzyme according to the average fragment size produced by each enzyme determined using *in silico* digestion of genomic DNA, step (2) evaluating the *in silico* frequency of fragment size distribution between individual chromosomes, step (3) selecting those enzymes that generate fragments with the majority between 100 bp and 3,000 bp, step (4) weighing the advantages and disadvantages of blunt-end sites vs. cohesive-end sites, step (5) elimination of methylation sensitive enzymes with methylation-insensitive isoschizomers, and step (6) elimination of enzymes with recognition sites within the binary vector sequence (T-DNA and plasmid backbone). Step (7) includes the selection of a second restriction enzyme with highest number of recognition sites within regions not covered by the first restriction enzyme. Step (8) considers primer and adapter sequence optimization, selecting the best adapter-primer pairs according to their hairpin/dimers and secondary structure. In step (9), the efficiency of genomic library development was improved by column-filtration of digested DNA to remove restriction enzyme and phosphatase enzyme, and most important, to remove small genomic fragments (<100 bp) lacking the T-DNA insertion, hence improving the chance of ligation between adapters and fragments harbouring a T-DNA. Two enzymes, *Nsi*I and *Nde*I, fit these criteria for the *Arabidopsis thaliana* genome. Their efficiency was assessed using 54 T_3_ lines from an Arabidopsis SK enhancer population. Over 70% success rate was achieved in amplifying the flanking sequences of these lines. This strategy was also tested with *Brachypodium distachyon* to demonstrate its applicability to other larger genomes.

## Introduction

The identification of flanking sequence tags (FST) has been used to determine the location of T-DNA insertion events in genomic DNA. This approach is often used to find new genes in populations developed through insertional mutagenesis (either T-DNA or transposable elements). Methods to obtain these FSTs include TAIL-PCR [1], inverse PCR [2], plasmid rescue [3] and genome walking [4]. Non-specific end products are the main drawback of TAIL-PCR due to degenerate primers being used in this method [5]. Inverse PCR and plasmid rescue are limited if suitable restriction enzyme recognition sites nearest to the T-DNA insertion site are outside the amplification range of *Taq* DNA polymerases.

Due to the use of specific primers in PCR reactions, genome walking has been one of the preferred approaches to identify flanking sequences in populations developed through insertional mutagenesis, especially in model plants such as *A. thaliana* [6], [7], [8], rice [9] and *Brachypodium distachyon* [10]. The success of this method relies on the presence of appropriate numbers of recognition sites for restriction enzymes used in generating genomic libraries. In addition, success depends on the efficient ligation of adapter sequence to the digested DNA, a reaction which is more efficient with the use of cohesive-end restriction digestion of genomic DNA. Different strategies have been suggested to overcome the above-mentioned shortfalls, including modified versions of adapters [11], [12], [13], biotinylated primers [14], touch-down PCR [15], [16], template blocking PCR [17], prevention of self-ligation through partial fill-in of digested DNA [18], dephosphorylation of 5′ ends [19], and incorporation of ddNTP at the 3′ end of digested fragments [20]. Despite the above efforts, genomic DNA should be digested by several restriction enzymes (cutting different region of the genome) to generate multiple genomic libraries. A survey of the literature shows efficiencies of 44.1% and 50% for *Brachypodium* and rice, respectively, when genome walking is the method for identifying flanking regions [10], [21].

Here, we describe a new method which depends heavily on determining the distribution of recognition sites for non-ambiguous palindromic restriction enzymes. We show that candidate restriction enzymes in genome walking should be selected according to an expanded set of criteria, including average fragment size produced after genomic DNA digestion, frequency of recognition sites within the genome, methylation sensitivity of restriction enzymes, and the presence of enzyme recognition sites within the T-DNA sequence. We also, provide other recommendations and have tested this method *in silico* and *in vivo* with *A. thaliana* mutant lines and *in silico* with *Brachypodium distachyon*.

## Materials and Methods

### Plant material and DNA preparation

Fifty-four (54) *Arabidopsis* T_3_ mutant lines harboring T-DNA insertion events from pSKIO15 (SK population developed at Saskatoon Research Centre) were tested in this study [6]. Genomic DNA extraction was carried out using the CTAB method [22].

### Screening to find suitable restriction enzymes for genomic library construction

Sequence data (TAIR10-assembly, Golden path length = 119 Mbp) for *A. thaliana* was obtained from The *Arabidopsis* Information Resource. Step (1), the number of recognition sites for 87 non-ambiguous palindromic enzymes was determined for each chromosome and the plastid and mitochondria genomes of *Arabidopsis* and *Brachypodium* after *in silico* digestion of their gDNA using Vector NTI V.11 (Invitrogen Co., Carlsbad, CA). step (2) data were collected based on “complete digestion” to simplify the process, then pooled to obtain the total number of fragments at the genome level. After *in silico* digestion, the resulting fragments for each enzyme were grouped by sizes distributed into three ranges: <100 bp, 100–3,000 bp, and >3000 bp in length. Step (3) restriction enzymes producing the highest percentage of average fragment sizes of 100–3000 bp were considered for further analysis and step (4), the (dis)advantage of blunt-end vs. cohesive-end sites were considered in choosing the candidate enzymes. This fragment size range (100–3000 bp) was selected as it is well within the amplification range of Taq polymerase under optimal conditions. Statistical analysis of the *in silico* digestion products was performed using SAS v9.1 (SAS Institute Inc., Cary, NC). Step (5), to limit the impact of incomplete digestion, enzymes sensitive to DNA methylation were avoided, or where possible, methylation-insensitive isoschizomers were selected in their place. Step (6), enzymes with recognition sites within T-DNA and binary plasmid backbone sequences were also excluded from the candidate enzymes. Step (7)m for situations where *in silico* fragments >3000 bp were produced by the first restriction enzyme, a second restriction enzyme was selected to cover these regions. Restriction sites for fragments >3000 bp (after digestion by the primary enzyme) were obtained for each chromosome and sequences for these fragments were retrieved from *Arabidopsis* genome using the Extractseq function in EMBOSS software package [23]. CLC Genomics 4.6 (CLC Bio Katrinebjerg, Denmark) was used to analyse *in silico* restriction digestion for the fragments produced by the first restriction enzyme. A custom Perl script was developed in a CLC output file to quickly calculate fragment sizes produced by *in silico* digestion of the secondary enzymes. Statistical analysis of the fragment frequencies was analysed using SAS. A custom Perl script combining these analyses was developed and is available upon request.

### Selection and modification of adapter and primers

Step (8), adapters from the Universal Genome Walker (UGW) kit (Clontech, Mountain View, CA), SWA [24] and ADP2 [10] and primers matching restriction enzymes which had passed through the evaluation process above were compared for secondary structures (including, hairpins and self-dimerization) using Oligoanalyzer (Integrated DNA Technologies Inc., U.S.A.) and OligoCalc [25] (Table 1). Alignments were performed using BlastN with selected primers and adapters against the *Arabidopsis* genome to ensure specificity of these sequences. The adapter sequence, CGCAGGCTGGCAGTCTCTTTAGGGTTACACGATTGCTT, described by Tsuchiya et al. [24] was modified to reflect the recognition sequences for *Nsi*I and *Nde*I. Reverse strand of adapter sequences (SWA-R-*Nsi*I and SWA-R-*Nde*I) were modified by amination at their 3′ end to prevent concatenation of adapter sequences and phosphorylation of their 5′ termini to enhance ligation reaction [24].

**Table 1:** List of oligonucleotides used for genome walking in *Arabidopsis* with restriction enzymes *Nsi*I and *NdeI*.

Oligo name	Oligo sequence (5′ = >3′)	Primer use
SWA-F-*Nsi*I	CGCAGGCTGGCAGTCTCTTTAGGGTTACACGATTGCTTTGCA	*Nsi*I adapter- forward strand
SWA-F-*Nde*I	CGCAGGCTGGCAGTCTCTTTAGGGTTACACGATTGCTT	*Nde*I adapter- forward strand
SWA-R-*Nsi*I	Phos-AAGCAATCGT GT-Amin group	*Nsi*I adapter- reverse strand
SWA-R-*Nde*I	Phos-TAAAGCAATCGT GT-Amin group	*Nde*I adapter- reverse strand
GW-F-out	CGCAGGCTGGCAGTCTCTTTAG	1° PCR
GW-F-in	TCTCTTTAGGGTTACACGATTGCTT	2° PCR
LB-R-out	GACAACATGTCGAGGCTCAGCAGGA	1° PCR
LB-R-in	TGGACGTGAATGTAGACACGTCG	2° PCR
LB-R-seq	ATACGACGGATCGTAATTTGTCG	sequencing

1° , denotes primary PCR reaction, 2°, denotes secondary nested PCR reaction.

### Preparation of 10× stock solution of adapters for *Arabidopsis*

*Nsi*I and *Nde*I adapters were prepared (Table 1) [24] by annealing forward and reverse strands specific for each enzyme (SWA-F-*Nsi*I/SWA-R-*Nsi*I and SWA-F-*Nde*I/SWA-R-*Nde*I). A 12.5 µl of 200 µM solution of forward and reverse strands for each adapter was mixed with 10 µl of NEBuffer 4 (10×) (New England Biolabs, Pickering, Ontario) and 64 µl of sterile ultrapure H_2_O in 250 µl PCR tubes. Using a PCR machine, adapters were annealed with one cycle of 94°C for 2 min, then synthesized at 70°C for 5 min and 37°C for 5 min, and stored at −20°C until further use. Adapter tubes were brought to 32°C prior to ligating them with genomic DNA.

### Preparation of adapter-ligated *Arabidopsis* genomic DNA

*Arabidopsis* Genomic DNA (500 ng) was digested with 10 units of either *Nsi*I or *Nde*I (NEB, Pickering, Ontario) in a final volume of 20 µl overnight at 37°C. Step (9), in preparation for adapter-ligation, digested DNA was treated with Antarctic phosphatase according to the manufacturer's instruction (NEB, Pickering, Ontario), filtered through PCR purification columns (Qiagen, Mississauga, Ontario), and diluted in 50 µl H_2_O. Prior to adapter ligation, column-filtered genomic fragments were heated to 50°C for 5 min to eliminate base-pairing between overhanging ends. Sample temperature was then reduced to 32°C and 2 µl of stock solution (25 µM) of enzyme-specific adapter was added to each tube. Ligation was performed at 25°C overnight by adding T4 DNA ligase and buffer (Invitrogen Co., Carlsbad, CA) according to the manufacturer's instructions in 60 µl final reaction volume.

### PCR amplification of the flanking regions in *A. thaliana* SK mutants

Primary PCR reactions contained 2 µl of 10× PCR buffer (Invitrogen), 2 µl of 2 mM deoxynucleotide triphosphates (dNTPs), 1.2 µl of MgCl_2_ (50 mM), 0.2 µl of Taq DNA polymerase (Invitrogen Co., Carlsbad, CA), 1 µl (10 mM) of SAP1 (first forward primer for adapter), 1 µl (10 mM) of LB-R-out (first reverse primer from left border of T-DNA insert) and 1 µl of adapter-ligated DNA (PCR template) in a total volume of 20 µl. Primers and adapters are listed in Table 1. PCR conditions were as follow: 94°C for 2 min, followed by 35 cycles of 94°C for 30 s, 60°C for 30 s and 72°C for 2 min, followed by one cycle of final extension at 72°C for 7 min. For subsequent nested PCR reactions, 1 µl of 100-fold diluted primary PCR product was used as a template and amplification followed the same steps as primary PCR, except that the annealing temperature was increased to 62°C and nested primers were used (Table 1). PCR products were visualized on 1% agarose gels in 1× TAE buffer. All visible bands were extracted from the gel using a Qiagen gel extraction kit. Sequencing was performed on these fragments using LB-R-seq primers (Table 1) and a 3730xl DNA Analyzer (Applied Biosystems, Carlsbad, Ca) at the Plant Biotechnology Institute, Saskatoon, SK, Canada.

## Results and Discussion

Genome Walking was developed to characterize flanking DNA regions from already known genomic regions or from mutations by T-DNA and transposon insertion [4]. However the efficiency of genome walking remains relatively low [10], [21] and restriction enzymes used for this approach have never been evaluated in relation to whole genome sequences for an individual plant species. The availability of whole genome sequence data for model species allows the genome walking protocol to be specifically optimized. Here we developed a methodology to determine the optimal restriction enzymes to use for genome walking according to the frequency and size of genomic fragments produced by these restriction enzymes.

### Criteria for choosing the best restriction enzyme(s) for genome walking

**Figure 1:**
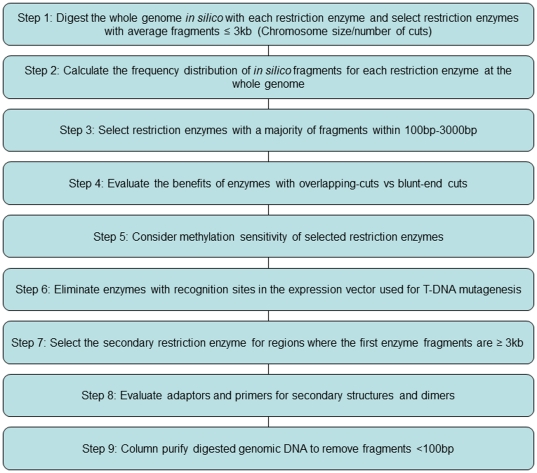
Flow chart outlining the steps used in optimized genome walking.

It has been assumed that the occurrence of restriction sites in a genome can be calculated by the simple mathematical formula [1/(4^N^)], where N is the number of nucleotides present in the recognition site [5], [26], [27]. The probability of this occurrence for enzymes in the classes of 4 bp recognition sites is 1/256 bp, of 6 bp sites is 1/4,096 bp, and of 8 bp sites is 1/65,536 bp. These calculations do not take into account the non-random arrangement of nucleotides within the genome. To address this deficiency, criteria were developed for selecting the most suitable enzymes to optimize genome walking (Figure 1). The frequency of enzyme recognition sites within the *Arabidopsis* genome was determined for 87 palindromic enzymes with single non-ambiguous restriction sites. Many of the enzymes showed frequencies with broad ranges outside the frequency range calculated for their specific restriction site class (Table S1; Figure S1 shows for *Nsi*I only). For example, when evaluating 4-bp enzymes in *Arabidopsis*, the number of restriction sites was 279,408 for *Bfa*I and 57,227 for *Gla*I. For 6-bp enzymes like *Dra*I and *Ssp*I, the 137,251 and 118,757 sites, respectively, are higher than the number of sites for *Gla*I. This skewed frequency strongly impacts the choice of restriction enzymes used in genome walking, and this test is the 1^st^ step (criterion) for consideration in restriction enzyme selection.

Twenty-nine restriction enzymes producing either blunt-ended fragments or overlapping-ended fragments and producing at least 39,000 fragments in the *A. thaliana* genome were then selected as candidate enzymes for fragment size distribution analysis. These enzymes produce fragment sizes ≤3,000 bp. Considering the possibility of genome walking from both ends of T-DNA molecule, the largest fragment required to be amplified is 1500 bp, which falls well within the amplification range of conventional *Taq* DNA polymerases under standard amplification conditions [28]. Fragment size within polymerase amplification range, therefore, is the 2^nd^ criterion when enzymes are selected for genome walking and is often overlooked. For example, the average fragment sizes produced by *in silico* digestion with *Dra*I, *Eco*RV, *Pvu*II and *Stu*I enzymes (from the Clonetech Genome Walker™ kit) for *Arabidopsis* are 0.9, 4, 6 and 12 kb, respectively (Table S1). Hence only one enzyme in this kit, *Dra*I, satisfied this important criterion in *Arabidopsis*.

Frequency distribution of genomic fragment sizes after *in silico* digestion of a whole genome and individual chromosomes was also evaluated as a 3^rd^ selection criterion for each restriction enzyme under consideration. To date, the choice of restriction enzymes for genome walking has been based either on the assumption of random distribution of restriction enzymes [5] or the digestion pattern of BAC clones from the given species, without consideration of fragment size distribution [10]. We evaluated genome-wide size distribution along each of the five Arabidopsis chromosomes (*Nsi*I in Figure S2) and for the Arabidopsis chloroplast and mitochondrial genomes (data not shown) for the 29 restriction enzymes with average fragment size <3000 bp in *Arabidopsis* (Table 2). In general, the percentage of fragments smaller than 100 bp should be considered when choosing the best candidate enzyme, since high levels of these small fragments could reduce the ligation efficiency between the adapter with larger fragments. As stated earlier, fragment sizes over 3000 bp also should be minimized (criterion 2). Among the cohesive-end cutter restriction enzymes tested, those with the best frequency distribution for genome walking in *Arabidopsis* were *Ase*I, *Bfa*I, *Hind*III, *Pab*I, SspI, *Tai*I, and *Taq*I, with 70% to 79% of their fragments within the 0.1–3 kb range (Table 2). Among blunt-end enzymes, *Dra*I, *Hae*III, *Psi*I, *Rsa*I, *Ssp*I may also be considered for genome walking in *A. thaliana*, since 71% to 79% of their fragments sizes fell within 0.1–3 kb (Table 2). Strong consideration should be given to using enzymes which generate cohesive ends, unless there is a very compelling advantage to using enzymes producing blunt fragment ends (4^th^ criterion). Despite the advantage of being able to use universal adapters with blunt-end restriction enzymes, cohesive-end restriction enzymes have a 10-fold higher ligation rate compared with blunt end enzymes [29], [30], and hence a much higher capacity to detect flanking regions in genome walking. This higher ligation rate can be a great advantage even though specific adapters are required for each cohesive-end restriction enzyme and a concomitant increase in labour to generate genome walking libraries. When possible, this drawback can also be negated by selecting cohesive-end restriction enzymes with compatible overhang-ends. If one decides to use blunt-end enzymes, then *Rsa*I, *Hae*III, *Ssp*I, *Psi*I and *Dra*I are better candidates for genome walking in *Arabidopsis*, as pointed out above. Our study is the first report presenting the importance of restriction enzyme fragment size distribution in genome walking and clearly demonstrates its importance at the whole genome and individual chromosome level.

**Table 2:** Fragment distribution frequency, methylation sensitivity and vector representation of 29 restriction enzymes with high numbers of fragments within a 100–3000 bp range in *Arabidopsis*.

Restriction enzyme	Fragments (%)			Methylation sensitive	Presence within vector pSKI015	Cohesive or blunt end
	<100 bp	0.1–3 kb	>3 kb			
*Alu*I	37.84	62.12	0.03	-	Y	B
*Ase*I	15.84	71.51	12.66	-	Y	C
*Bfa*I	23.54	76.27	0.19	-	Y	C
*Bgl*II	5.84	61.79	32.37	-	Y	C
*Bsp*HI	5.02	63.35	31.64	Dam	Y	C
*Bst*UI	12.37	63.88	23.74	CG	Y	B
*Cha*I	34.83	65.13	0.03	?	Y	C
*Dpn*I	34.83	65.13	0.03	Dam	Y	B
*Dra*I	22.53	71.49	5.98	-	Y	B
*Fat*I	33.69	66.30	0.01	-	Y	C
*Gla*I	9.04	67.04	23.91	-*	Y	B
*Hae*III	17.07	76.75	6.17	-	Y	B
*Hha*I	9.04	67.04	23.91	CG	Y	C
*Hind*III	8.29	72.72	18.99	-	Y	C
*Hin*P1I	9.04	67.04	23.91	-	Y	C
*Hpa*II	25.06	69.04	5.90	CG	-	B
*Mbo*I	34.83	65.14	0.03	Dam, CG	Y	C
*Mse*I	61.55	38.45	0.00	-	-	B
*Nde*I	4.99	59.09	35.93	-	-	C
*Nla*III	33.69	66.30	0.01	-	Y	C
*Nsi*I	5.76	63.88	30.37	-	-	C
*Pab*I	20.39	79.13	0.48	?	Y	C
*Pci*I	5.47	64.44	30.08	-	Y	C
*Psi*I	13.35	74.35	11.69	-	Y	B
*Rsa*I	20.39	79.13	0.48	CG	Y	B
*Sel*I	12.37	63.88	23.74	CG	-	C
*Ssp*I	17.21	75.12	7.67	-	Y	B
*Tai*I	22.73	76.47	0.80	CG	Y	C
*Taq*I	28.95	70.74	0.31	Dam	Y	C

* *Gla*I is a methylation dependent endonuclease which only cleaves DNA when 5-methylcytosine or 5-hydroxymethylcytosine lies within its recognition sequence [34].

? information not available; Y, yes; B, blunt; C, cohesive.

Although *Pab*I, *Tai*I, *Bfa*I, *Hind*III, *Ase*I and *Taq*I were selected as the best frequency distribution candidates for *Arabidopsis* amongst enzymes generating cohesive ends, the methylation sensitivity of *Tai*I and *Taq*I potentially reduces the probability of generating fragments within the optimal size range for genome walking (Table 2). Methylation sensitivity of blunt-ended enzymes, eg. *Eco*RV (CpG) and *Stu*I (Dcm) from the Genome Walker™ kit, also reduces their potential utility in genome walking, and from our evaluation these two enzymes now show three limitations for *Arabidopsis*. Depending on their availability, isoschizomers may be used for these restriction enzymes to reduce the problems associated with methylation sensitivity; for example, *Rsa*I can be replaced by M.*Rsa*I. These examples highlight methylation sensitivity as the 5^th^ criterion to consider when selecting restriction enzymes for genome walking.

Plasmid backbone sequence can be transferred along with the T-DNA into the plant genome following imprecise processing of the border repeats [31]. Therefore, the presence of enzyme recognition sites within a binary vector sequence was the 6^th^ criterion we investigated when evaluating candidate restriction enzymes for insert populations. Due to the potential for larger fragments arising from insertion events, this phenomenon could reduce genome walking efficiency. Among the enzymes that generate fragments with cohesive ends and result in a high percentage of fragments within 100–3000 bp (Table S1), *Ase*I, *Bfa*I, *Bgl*II, *Bsp*HI, *Hind*III, *Pci*I and *Pab*I had at least one recognition site within the pSKI015 vector sequence, which was the vector used to generate several mutant populations in Arabidopsis [5], and consequently, these enzymes are less useful for these populations. The enzymes *Nsi*I and *Nde*I possessing 64% and 59% of genomic fragments within the 100–3000 bp size range, respectively, are the only two enzymes with cohesive-ends and no recognition sites within the pSKI015 vector sequence (Figure S3 shown for *Nsi*I). Due to *in silico* digestion resulting in a higher percentage of fragments within 100–3000 bp, *Nsi*I was selected as the primary candidate enzyme for genome walking for this species when using pSKIO15 as the T-DNA source.

As the 7^th^ criterion, secondary restriction enzymes should be selected for genome walking to include maximum number of recognition sites within fragments ≥3 kb from *in silico* digestion with primary restriction enzyme. In order to achieve this goal, *in silico Nsi*I-digested fragments ≥3 kb were re-digested *in silico* with other candidate enzymes satisfying previous selection criteria (ie: cohesive ending, highest fragment proportions within 100–3000 bp, methylation sensitivity, and no recognition sites within the vector). The examination of the fragments >3000 bp resulting from the *Nsi*I digest were subjected to *in silico* digestion and fragment size distribution for six of those enzymes is presented in Table 3. Here, the enzyme *Pci*I has the highest number of fragments within 100–3000 bp range. Considering sites within the pSKIO15 vector and the *Arabidopsis* SK population, only *Nde*I fulfilled the 7^th^ criteria with 73% of its fragments being within the required 100–3000 bp range.

**Table 3:** Fragment distribution frequency for *in silico Nsi*I-digested fragments ≥3000 bp after *in silico* digestion with second restriction enzyme.

Secondary Restriction enzyme	Fragments (%)		
	<100 bp	100–3000 bp	>3000 bp
BfaI	24.78	75.11	0.11
ChaI	36.32	63.66	0.02
NdeI	6.67	72.89	20.45
PciI	6.59	76.59	16.82
SelI	13.52	74.30	12.18
TaiI	23.79	75.72	0.49

### Other Improvements for Genome Walking

The 8^th^ criterion (adapter and primer evaluation) is dependent on the set of enzymes which successfully came through the first 7 steps of enzyme selection. Here, the palindromic nature of primers and adapters should be considered. A number of different adapters have been suggested for genome walking, including the Clontech GenomeWalker^tm^ Kit universal adapter for blunt end ligation and adapters used by several groups for enzymes producing fragments with overlapping ends [5], [10], [24]. After narrowing down the list of restriction enzymes for *Arabidopsis*, adapters and primers (including *Nsi*I and *Nde*I recognition sequences) were compared for any possible secondary structure issues, including hairpins and self-dimerization and adapter/primer homology with *Arabidopsis* genomic DNA (Table 4). BlastN analysis showed that these oligos had no homology with Arabidopsis genomic sequence.

**Table 4:** Adapter and primer sets evaluated in this study. Hairpin and self-dimer structures for each oligo were measured by Oligoanalyzer and OligoCalc.

Adapter	Sequence	Reference	ΔG hairpins kcal.mole	ΔG self-dimers kcal.mole
GW. Adp	GTAATACGACTCACTATAGGGCACGCGTGGTCGACGGCCCGGGCTGGT	Clontech	−4.28	−22.17
AP1	GTAATACGACTCACTATAGGGC	Clontech	0.65	−6.59
AP2	ACTATAGGGCACGCGTGGT'	Clontech	−0.36	−16.95
SWA-F	CGCAGGCTGGCAGTCTCTTTAGGGTTACACGATTGCTT	[24]	−0.73	−5.09
SAP1	CGCAGGCTGGCAGTCTCTTTAG	[24]	−0.5	−3.61
SAP2	CTCTTTAGGGTTACACGATTGCTT	[24]	0.58	−3.61
ADP2	CTAATACGACTCACTATAGGGCTCGAGCGGCCGGGCAGGT	[10]	−2.29	−16.5
AP1	GGATCCTAATACGACTCACTATAGGGC	[10]	−0.8	−10.76
AP2	TATAGGGCTCGAGCGGC	[10]	−0.56	−16.24

Prior to the construction of genomic libraries for genome walking, another step also was included (the 9th criterion), in which gDNA fragments from restriction enzyme digestion and phosphorylation are filtered through PCR purification columns prior to adapter ligation. This filtration step not only removes restriction enzymes and phosphatase enzymes; more important, it also removes small genomic fragments (<100 bp) that might participate in concatenation reactions. Hence, for T-DNA insertion populations, this step removes small fragments without a T-DNA and increases the chance of ligation between adapters and longer fragments harboring a T-DNA insert, and thus improves the efficiency of genome walking.

### Confirmation of *in silico* criteria using Arabidopsis SK lines

**Figure 2:**
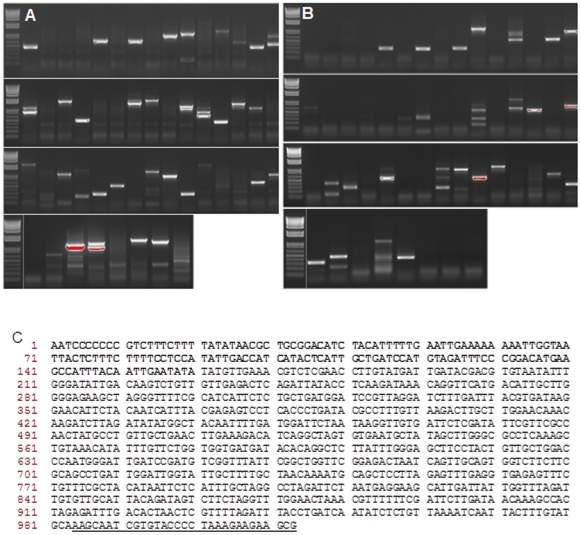
Identification of T-DNA flanking sequence in 54 *A. thaliana* lines by genome walking. PCR amplification of sequences flanking the left border of T-DNA inserts (A) *Nsi*I digested DNA. (B) *Nde*I digested DNA. The first lane for each row is 1 kb plus ladder (Invitrogen). (C) Example of T-DNA flanking sequence obtained from transgenic line p416 from the *A. thaliana* SK population. In this example, T-DNA was inserted into the *TTG1* gene. The T-DNA footprint is highlighted in bold and the GW-adapter sequence is underlined.

The outcome of the expanded *in silico* selection method was tested by conducting genome walking using 54 *Arabidopsis* T_3_ enhancer lines of the SK population. Each of these lines arose from independent T-DNA insertion events. Using the expanded criteria, we selected the primary restriction enzyme *Nsi*I and the secondary enzyme *Nde*I. Both produce cohesive-end fragments and are insensitive to methylation. PCR products resulting from these lines (Figure 2 A, B) were purified and sequenced directly (without further cloning) to confirm whether the amplified fragment represented a targeted flanking sequence (FST) with the T-DNA sequence on one side and the adapter signature at the other end. This is illustrated for the SK line P416, in which the T-DNA is inserted into the *TRANSPARENT TESTA GLABRA1* (*TTG1*) gene (Figure 2 C). In some cases, two fragments with similar sizes were amplified together and the obtained sequence had the T-DNA sequence signature while the remainder of the sequence had no match in *A. thaliana* genome (Figure S3). For these cases, an additional cloning step was included to separate the fragments and to identify the flanking sequences.

When *Nsi*I and *Nde*I enzymes were used with the other optimized methods, we were able to identify 70% of the flanking regions from the left-border of T-DNA in *Arabidopsis*. This is much higher than reported previously for genome walking performed within mutagenized populations of rice (50%) and *B. distachyon* (44.1%) [10], [21].

The ‘one enzyme-two border’ approach [10], which uses only one enzyme to conduct genome walking from both the right- and left-borders of the T-DNA and which has been tested on *Brachypodium* [10] was also tested on the right border of *Arabidopsis* lines harboring the T-DNA inserts from pSKIO15 vector. However the success rate for flanking sequence identification was less than 5% (data not shown) from the right border in these lines. The inability of the ‘one enzyme-two border’ approach with SK lines could be due to incomplete insertion of regions near the T-DNA right-border, as verified for the pSKIO15 vector [32], or potentially due to head-to-head tandem repeats close to the right border, known to be an issue in rice using different vector [21]. Hence with the SK population or other populations developed by this vector, two or more additional enzymes should be used to amplify the flanking region from the left border.

The same expanded *in silico* approach could be followed when choosing restriction enzymes to conduct genome walking studies for any organism with available genome sequence. Hence, we also screened the *B. distachyon* genome for the frequency of recognition sites for different restriction enzymes. After *in silico* digestion of this genome, restriction enzymes specifying cohesive ends and insensitive to DNA methylation were evaluated (Table S2). The enzymes, *Fat*I, *Nla*III, *Mse*I, *Cha*I, *Bfa*I, *Pab*I, *Sel*I, *Nsi*I, *Pci*I, *Ase*I, *Sph*I, *Pst*I, *Nco*I produced fragments with an average size of less than 3000 bp. In an earlier study, *Bfa*I had been used as the candidate restriction enzyme in *B. distachyon* FST identification due to the small fragment sizes produced (<500 bp) following restriction analysis of its BAC library [10]. In the current study, *in silico* digestion of the *B. distachyon* genome with *Bfa*I resulted in fragments with an average size of 336 bp (Table S2), and 69% of fragments within 100–3000 bp size range. However, the distribution was strongly skewed toward small fragments with 12.5% of the fragments between 50–100 bp and 17.5% less than 50 bp (Figure S1B). Due to the increased probability of self-ligation between these small fragments, the ligation efficiency between adapter and target fragments is likely to be reduced when libraries made using *Bfa*I digest. In addition, *Bfa*I digestion might result in ligation of multiple small fragments (concatenation reaction) between the T-DNA and adapter sequence. These findings may explain why Thole et al. (2009) were able to identify only 50% of T-DNA flanking regions in *B. distachyon* using *Bfa*I restriction enzyme [10].

### Conclusion

In this study, a new method for selecting candidate restriction enzymes in genome walking was developed and tested in two genomes. The method features an expanded set of criterion for enzyme selection, as well as a optimizing filtration step. This method will be useful as a guideline for genome walking in species in which genomes are sequenced or populations developed by insertional mutagenesis. We have tested this method for genome walking. However new genomic techniques like reduced-representation libraries (RRLs), restriction-associated DNA sequencing (RAD-seq) and multiplexed shotgun genotyping (MSG), [33] which all rely on restriction enzyme digestion, can also benefit from this strategy.

## Supporting Information

Figure S1 **Fragment distribution produced by the** ***A. thaliana*** **and** ***Brachypodium distachycon*** **genomes following** ***in silico*** **digestion of gDNA. (A)** ***Arabidopsis*** **digested with** ***Nsi*** **I showing 97.3% of the genome. (B)** ***B. distachyon*** **digested with** ***Bfa*** **I showing 99.9% of the genome.**
(TIF)

Figure S2 **Genomic fragment size distribution along each of five** ***A. thaliana*** **chromosomes after** ***in silico*** **digestion with** ***Nsi*** **I.**
(TIF)

Figure S3 **Sequencing chromatograph for two fragments that were amplified together from the** ***Arabidopsis*** **SK population and sharing the same T-DNA signature at the 5′ end (double end arrow).**
(TIF)

Table S1 Number of fragments produced for each *A. thaliana* chromosome by *in silico* digestion using non-ambiguous, palindromic restriction enzymes.
(DOCX)

Table S2 Number of fragments produced for each *Brachypodium distachyon* chromosome by *in silico* digestion using non-ambiguous, palindromic restriction enzymes.
(DOCX)
