# Supplementary figures and images for: Revised Selection Criteria for Candidate Restriction Enzymes in Genome Walking
Source: PLoS One. 2012 Apr 11;7(4):e35117. doi: 10.1371/journal.pone.0035117 (PMC3324424; doi:10.1371/journal.pone.0035117)

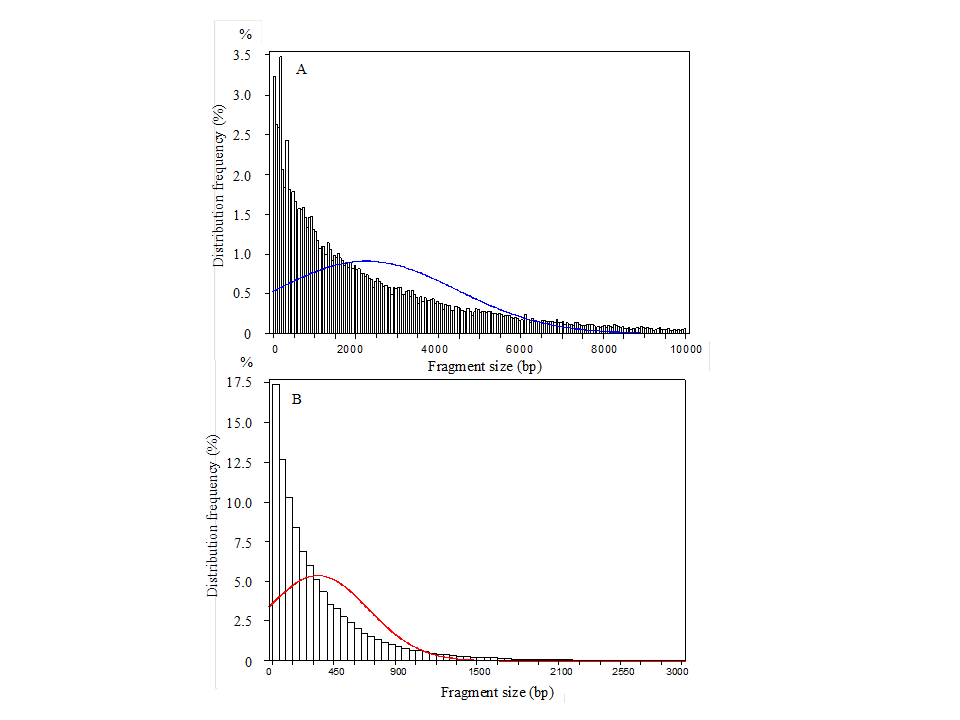

Supplement: Figure S1 — Fragment distribution produced by the A. thaliana and Brachypodium distachycon genomes following in silico digestion of gDNA. (A) Arabidopsis digested with Nsi I showing 97.3% of the genome. (B) B. distachyon digested with Bfa I showing 99.9% of the genome. (TIF) [file pone.0035117.s001.tif]

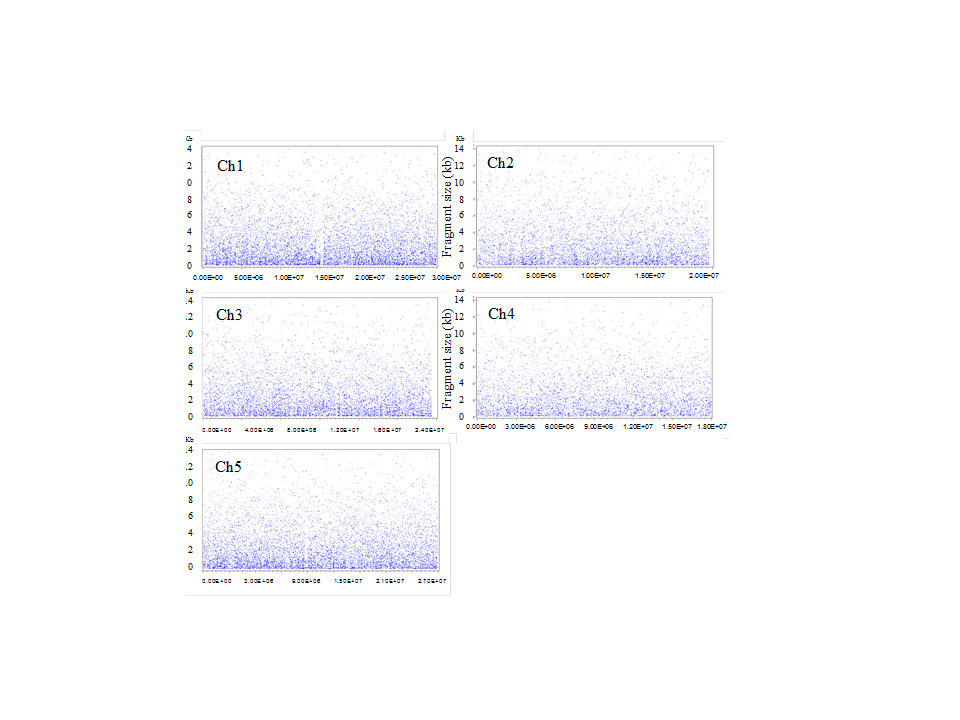

Supplement: Figure S2 — Genomic fragment size distribution along each of five A. thaliana chromosomes after in silico digestion with Nsi I. (TIF) [file pone.0035117.s002.tif]

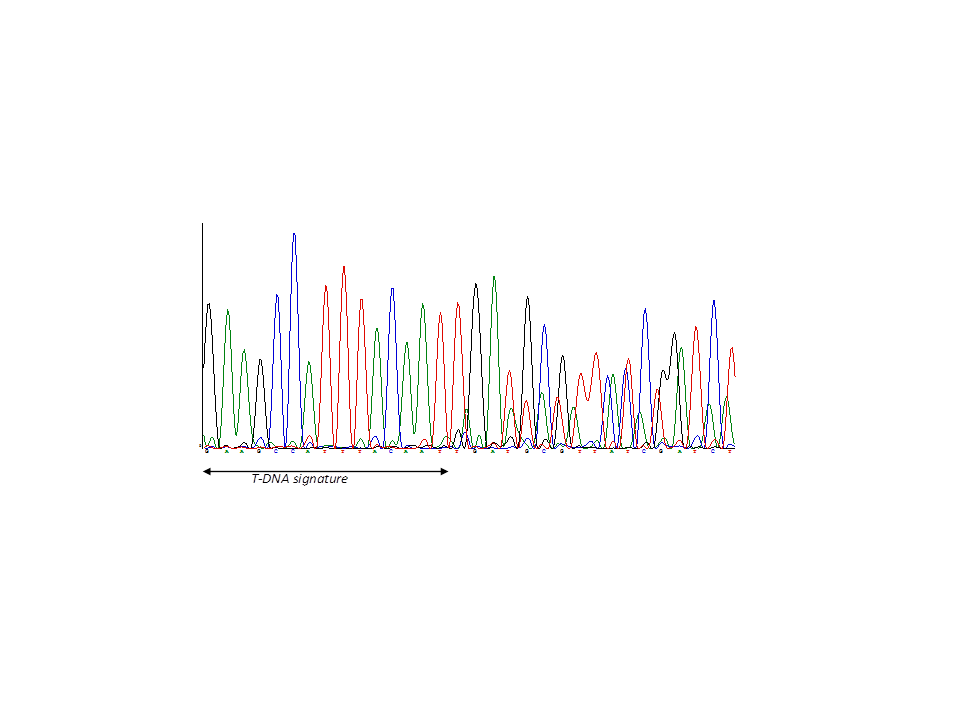

Supplement: Figure S3 — Sequencing chromatograph for two fragments that were amplified together from the Arabidopsis SK population and sharing the same T-DNA signature at the 5′ end (double end arrow). (TIF) [file pone.0035117.s003.tif]
