# Supplementary material for: Revised Selection Criteria for Candidate Restriction Enzymes in Genome Walking
Source: PLoS One. 2012 Apr 11;7(4):e35117. doi: 10.1371/journal.pone.0035117 (PMC3324424; doi:10.1371/journal.pone.0035117)
Supplement: Table S1 — Number of fragments produced for each A. thaliana chromosome by in silico digestion using non-ambiguous, palindromic restriction enzymes. (DOCX) [file pone.0035117.s004.docx]

| Restriction Enzyme | Ch1 (30Mb) | Ch2 (19.6Mb) | Ch3 (23.5Mb) | Ch4 (18.6Mb) | Ch5 (27Mb) | Plastid (154Kb) | Mitoch. (367Kb) | Total fragments in genome | Fragment Size (kb) |
| --- | --- | --- | --- | --- | --- | --- | --- | --- | --- |
| AatII | 2328 | 1582 | 1952 | 1409 | 1973 | 7 | 33 | 9284 | 12.9 |
| Acc65I | 2103 | 1448 | 1660 | 1308 | 1846 | 11 | 52 | 8428 | 14.2 |
| AcII | 7014 | 4609 | 5512 | 4265 | 6453 | 12 | 33 | 27898 | 4.3 |
| AfeI | 1205 | 809 | 944 | 743 | 1025 | 4 | 55 | 4785 | 25.0 |
| AflII | 7419 | 4948 | 5916 | 4805 | 6637 | 13 | 79 | 29817 | 4.0 |
| AgeI | 3777 | 2457 | 2988 | 2307 | 3338 | 17 | 48 | 14932 | 8.0 |
| AluI | 122411 | 77346 | 96094 | 76930 | 109071 | 453 | 2078 | 484383 | 0.2 |
| ApaI | 656 | 450 | 564 | 399 | 615 | 12 | 57 | 2753 | 43.5 |
| ApaLI | 2164 | 1225 | 1658 | 1315 | 1933 | 5 | 34 | 8334 | 14.4 |
| AseI | 23672 | 15853 | 17666 | 13885 | 20876 | 155 | 98 | 92205 | 1.3 |
| AvrII | 1754 | 1189 | 1362 | 1146 | 1595 | 18 | 65 | 7129 | 16.8 |
| BamHI | 4163 | 2892 | 3609 | 2654 | 3897 | 63 | 92 | 17370 | 6.9 |
| BbeI | 842 | 538 | 694 | 541 | 803 | 4 | 36 | 3458 | 34.6 |
| BcII | 13322 | 8443 | 10448 | 8079 | 11701 | 57 | 115 | 52165 | 2.3 |
| BfaI | 70091 | 46202 | 54886 | 44200 | 62273 | 374 | 1382 | 279408 | 0.4 |
| BglII | 11133 | 7276 | 8693 | 6916 | 9697 | 77 | 165 | 43957 | 2.7 |
| BmtI | 2582 | 1627 | 1965 | 1693 | 2207 | 5 | 105 | 10184 | 11.8 |
| BsiWI | 1657 | 1019 | 1217 | 1032 | 1354 | 6 | 40 | 6325 | 18.9 |
| BspEI | 3175 | 2022 | 2377 | 1989 | 2671 | 30 | 75 | 12339 | 9.7 |
| BspHI | 11420 | 7492 | 8971 | 6873 | 10131 | 40 | 70 | 44997 | 2.7 |
| BsrGI | 7538 | 4839 | 5740 | 4692 | 6466 | 35 | 47 | 29357 | 4.1 |
| BSSHII | 250 | 178 | 238 | 172 | 229 | 4 | 32 | 1103 | 108.5 |
| BstBI | 8289 | 5482 | 6858 | 5167 | 7799 | 90 | 128 | 33813 | 3.5 |
| BstUI | 14422 | 9657 | 11982 | 9277 | 13077 | 128 | 491 | 59034 | 2.0 |
| BstZ17I | 5172 | 3272 | 3982 | 3117 | 4647 | 24 | 52 | 20266 | 5.9 |
| ChaI | 108946 | 70660 | 87650 | 68199 | 97499 | 716 | 1660 | 435330 | 0.3 |
| ClaI | 7926 | 5186 | 6339 | 4782 | 7073 | 72 | 114 | 31492 | 3.8 |
| DpnI | 108946 | 70660 | 87650 | 68199 | 97500 | 716 | 1660 | 435331 | 0.3 |
| DraI | 34167 | 24212 | 26241 | 21140 | 31318 | 87 | 86 | 137251 | 0.9 |
| EagI | 843 | 652 | 659 | 469 | 763 | 9 | 43 | 3438 | 34.8 |
| Eco53kI | 5031 | 3346 | 4418 | 3351 | 4531 | 18 | 102 | 20797 | 5.8 |
| EcoRI | 9200 | 6105 | 7517 | 5658 | 8332 | 104 | 141 | 37057 | 3.2 |
| EcoRV | 8066 | 5074 | 6249 | 4859 | 7183 | 47 | 74 | 31552 | 3.8 |
| FatI | 114466 | 74829 | 90052 | 71033 | 101309 | 469 | 915 | 453073 | 0.3 |
| FspI | 1392 | 938 | 1122 | 902 | 1249 | 7 | 15 | 5625 | 21.3 |
| GlaI | 14098 | 9452 | 11477 | 8903 | 12559 | 112 | 626 | 57227 | 2.1 |
| HaeIII | 29926 | 20432 | 24617 | 19014 | 27170 | 202 | 871 | 122232 | 1.0 |
| HhaI | 14098 | 9452 | 11477 | 8903 | 12559 | 112 | 626 | 57227 | 2.1 |
| HindIII | 16963 | 10475 | 12570 | 11057 | 15776 | 46 | 170 | 67057 | 1.8 |
| HinP1I | 14098 | 9452 | 11477 | 8903 | 12559 | 112 | 626 | 57227 | 2.1 |
| HpaI | 6391 | 4368 | 5066 | 3994 | 5990 | 20 | 53 | 25882 | 4.6 |
| HpaII | 34441 | 22681 | 27521 | 21529 | 31497 | 245 | 1001 | 138915 | 0.9 |
| KasI | 842 | 538 | 694 | 541 | 803 | 4 | 36 | 3458 | 34.6 |
| KpnI | 2103 | 1448 | 1660 | 1308 | 1846 | 11 | 52 | 8428 | 14.2 |
| MboI | 108946 | 70660 | 87650 | 68199 | 97500 | 716 | 1660 | 435331 | 0.3 |
| McaTI | 250 | 178 | 238 | 172 | 229 | 4 | 32 | 1103 | 108.5 |
| MfeI | 9378 | 6254 | 7328 | 5678 | 8393 | 72 | 98 | 37201 | 3.2 |
| MluI | 1332 | 816 | 1128 | 821 | 1221 | 9 | 17 | 5344 | 22.4 |
| MscI | 4125 | 2905 | 3222 | 2600 | 3770 | 13 | 47 | 16682 | 7.2 |
| MseI | 247225 | 164236 | 186485 | 149220 | 221198 | 999 | 1238 | 970601 | 0.1 |
| NaeI | 1124 | 763 | 957 | 617 | 978 | 7 | 38 | 4484 | 26.7 |
| NarI | 842 | 538 | 694 | 541 | 803 | 4 | 36 | 3458 | 34.6 |
| NcoI | 5329 | 3466 | 4179 | 3343 | 4928 | 29 | 68 | 21342 | 5.6 |
| NdeI | 10086 | 6714 | 7634 | 6167 | 8961 | 32 | 59 | 39653 | 3.0 |
| NgoMIV | 1124 | 763 | 957 | 617 | 978 | 7 | 38 | 4484 | 26.7 |
| NheI | 2582 | 1627 | 1965 | 1693 | 2207 | 5 | 105 | 10184 | 11.8 |
| NlaIII | 114466 | 74829 | 90052 | 71033 | 101309 | 469 | 915 | 453073 | 0.3 |
| NruI | 1423 | 928 | 1139 | 875 | 1269 | 18 | 41 | 5693 | 21.0 |
| NsiI | 12762 | 7620 | 9129 | 7248 | 10561 | 47 | 81 | 47448 | 2.5 |
| PabI | 59917 | 38823 | 47017 | 37300 | 52876 | 331 | 825 | 237089 | 0.5 |
| PciI | 11902 | 7888 | 9168 | 7555 | 10593 | 34 | 34 | 47174 | 2.5 |
| PmlI | 3642 | 2322 | 2894 | 2354 | 3296 | 12 | 24 | 14544 | 8.2 |
| PsiI | 241140 | 15564 | 17562 | 14028 | 20708 | 90 | 94 | 309186 | 0.4 |
| PspOMI | 656 | 450 | 564 | 399 | 615 | 12 | 57 | 2753 | 43.5 |
| PstI | 5589 | 3350 | 4433 | 3443 | 4983 | 9 | 68 | 21875 | 5.5 |
| PvuI | 2440 | 1686 | 2083 | 1771 | 2266 | 10 | 63 | 10319 | 11.6 |
| PvuII | 4962 | 3104 | 4061 | 3182 | 4405 | 13 | 106 | 19833 | 6.0 |
| RsaI | 59917 | 38823 | 47017 | 37300 | 52876 | 331 | 825 | 237089 | 0.5 |
| SacI | 5031 | 3346 | 4418 | 3351 | 4531 | 18 | 102 | 20797 | 5.8 |
| SacII | 1066 | 738 | 838 | 668 | 959 | 10 | 31 | 4310 | 27.8 |
| SalI | 2370 | 1615 | 1922 | 1562 | 2239 | 11 | 40 | 9759 | 12.3 |
| ScaI | 5966 | 3875 | 4865 | 3810 | 5419 | 23 | 64 | 24022 | 5.0 |
| SciI | 4383 | 2824 | 3467 | 2760 | 3937 | 21 | 80 | 17472 | 6.8 |
| SelI | 14422 | 9657 | 11982 | 9277 | 13077 | 128 | 491 | 59034 | 2.0 |
| SfoI | 842 | 538 | 694 | 541 | 803 | 4 | 36 | 3458 | 34.6 |
| SmaI | 718 | 505 | 565 | 459 | 784 | 11 | 77 | 3119 | 38.4 |
| SnaBI | 4812 | 3010 | 3580 | 2733 | 4115 | 26 | 58 | 18334 | 6.5 |
| SpeI | 6380 | 4050 | 4828 | 3878 | 5689 | 17 | 99 | 24941 | 4.8 |
| SphI | 3249 | 2175 | 2621 | 2109 | 2885 | 12 | 49 | 13100 | 9.1 |
| SspI | 30104 | 20428 | 22742 | 18114 | 27139 | 149 | 81 | 118757 | 1.0 |
| StuI | 2519 | 1636 | 2069 | 1676 | 2235 | 25 | 75 | 10235 | 11.7 |
| TaiI | 58460 | 37578 | 45359 | 35474 | 51328 | 251 | 539 | 228989 | 0.5 |
| TaqI | 85683 | 56684 | 68945 | 55348 | 79069 | 672 | 1495 | 347896 | 0.3 |
| XbaI | 8137 | 5342 | 6572 | 5182 | 7296 | 44 | 79 | 32652 | 3.7 |
| XhoI | 4383 | 2824 | 3467 | 2760 | 3937 | 21 | 80 | 17472 | 6.8 |
| XmaI | 718 | 505 | 656 | 459 | 784 | 11 | 77 | 3210 | 37.3 |
| ZraI | 2328 | 1582 | 1952 | 1409 | 1973 | 7 | 33 | 9284 | 12.9 |

Ch, denotes chromosome and Mitoch. denotes mitochondria.
