# Supplementary material for: Revised Selection Criteria for Candidate Restriction Enzymes in Genome Walking
Source: PLoS One. 2012 Apr 11;7(4):e35117. doi: 10.1371/journal.pone.0035117 (PMC3324424; doi:10.1371/journal.pone.0035117)
Supplement: Table S2 — Number of fragments produced for each Brachypodium distachyon chromosome by in silico digestion using non-ambiguous, palindromic restriction enzymes. (DOCX) [file pone.0035117.s005.docx]

| Restriction Enzyme | Bd1 | Bd2 | Bd3 | Bd4 | Bd5 | Total fragments in  genome | Fragment size (kb) | Methylation sensitivity |
| --- | --- | --- | --- | --- | --- | --- | --- | --- |
|  | (75 Mb) | (60 Mb) | (60 Mb) | (948 Mb) | (28 Mb) |  |  |  |
| AatII | 11062 | 8886 | 8942 | 7191 | 4443 | 40524 | 6.7 | CpG |
| Acc65I | 10519 | 8504 | 8364 | 6839 | 4144 | 38370 | 7.1 | Dcm, CpG |
| AclI | 8431 | 6591 | 6669 | 5338 | 3193 | 30222 | 9.0 | CpG |
| AfeI | 8055 | 6510 | 6555 | 5006 | 3232 | 29358 | 9.3 | CpG |
| AflII | 10998 | 8810 | 8809 | 7136 | 4241 | 39994 | 6.8 | - |
| AgeI | 10446 | 8443 | 8531 | 7105 | 4182 | 38707 | 7.0 | CpG |
| AluI | 321235 | 253800 | 257537 | 207851 | 121315 | 1161738 | 0.2 | - |
| ApaI | 12417 | 10033 | 9832 | 8367 | 5155 | 45804 | 5.9 | Dcm, CpG |
| ApaLI | 17586 | 14201 | 13845 | 11136 | 6737 | 63505 | 4.3 | CpG |
| AseI | 32421 | 25408 | 25862 | 22002 | 11260 | 116953 | 2.3 | - |
| AvrII | 10146 | 8140 | 8888 | 7032 | 4112 | 38318 | 7.1 | CpG |
| BamHI | 16299 | 12901 | 13126 | 11140 | 6510 | 59976 | 4.5 | - |
| BbeI | 24548 | 19480 | 19128 | 15350 | 9453 | 87959 | 3.1 | ? |
| BclI | 26415 | 21136 | 21316 | 17263 | 10004 | 96134 | 2.8 | Dam |
| BfaI | 227019 | 177089 | 182870 | 148762 | 84876 | 820616 | 0.3 | - |
| BglII | 20759 | 16411 | 16598 | 13494 | 7748 | 75010 | 3.6 | - |
| BmtI | 20817 | 16260 | 16969 | 13238 | 7689 | 74973 | 3.6 | - |
| BsiWI | 11337 | 9189 | 8685 | 6493 | 4123 | 39827 | 6.8 | CpG |
| BspEI | 12444 | 9395 | 10025 | 8152 | 4822 | 44838 | 6.1 | Dam, CpG |
| BspHI | 24923 | 19312 | 19576 | 16187 | 9279 | 89277 | 3.0 | Dam |
| BsrGI | 22911 | 18013 | 18061 | 15082 | 8328 | 82395 | 3.3 | - |
| BssHII | 21928 | 17859 | 17387 | 13641 | 8837 | 79652 | 3.4 | CpG |
| BstBI | 12426 | 9768 | 9871 | 8016 | 4675 | 44756 | 6.1 | CpG |
| BstUI | 199205 | 163018 | 159095 | 124218 | 81661 | 727197 | 0.4 | CpG |
| BstZ17I | 11079 | 8594 | 8680 | 6977 | 3920 | 39250 | 6.9 | CpG |
| ChaI | 291066 | 231174 | 232667 | 189434 | 111102 | 1055443 | 0.3 | ? |
| ClaI | 21099 | 16916 | 16841 | 13442 | 7926 | 76224 | 3.6 | Dam, CpG |
| DpnI | 291066 | 231174 | 232667 | 189434 | 111102 | 1055443 | 0.3 | CpG |
| DraI | 33200 | 26014 | 26949 | 22056 | 12572 | 120791 | 2.3 | - |
| EagI | 25473 | 20434 | 20440 | 15612 | 9922 | 91881 | 3.0 | CpG |
| Eco53kI | 23441 | 18743 | 19179 | 15464 | 9113 | 85940 | 3.2 | CpG |
| EcoRI | 17898 | 14241 | 14276 | 11579 | 7008 | 65002 | 4.2 | CpG |
| EcoRV | 14777 | 11301 | 11697 | 9635 | 5558 | 52968 | 5.1 | CpG |
| FatI | 418130 | 330192 | 334785 | 273289 | 156669 | 1513065 | 0.2 | - |
| FspI | 10559 | 8807 | 8870 | 6757 | 4256 | 39249 | 6.9 | CpG |
| GlaI | 233279 | 190627 | 187800 | 146664 | 94857 | 853227 | 0.3 | ? |
| HaeIII | 274332 | 219946 | 219270 | 177521 | 107621 | 998690 | 0.3 | - |
| HhaI | 233279 | 190627 | 187800 | 146664 | 94857 | 853227 | 0.3 | CpG |
| HindIII | 23888 | 18734 | 19034 | 15455 | 9026 | 86137 | 3.2 | - |
| HinP1I | 233279 | 190627 | 187800 | 146664 | 94857 | 853227 | 0.3 | CpG |
| HpaI | 11439 | 9043 | 8952 | 7301 | 4235 | 40970 | 6.6 | CpG |
| HpaII | 256856 | 205724 | 207116 | 168322 | 104187 | 942205 | 0.3 | CpG |
| KasI | 24548 | 19480 | 19128 | 15350 | 9453 | 87959 | 3.1 | CpG |
| KpnI | 10519 | 8504 | 8364 | 6839 | 4144 | 38370 | 7.1 | - |
| MboI | 291066 | 231174 | 232667 | 189434 | 111102 | 1055443 | 0.3 | Dam, CpG |
| McaTI | 21928 | 17859 | 17387 | 13641 | 8837 | 79652 | 3.4 | ? |
| MfeI | 21821 | 16836 | 17430 | 13912 | 8092 | 78091 | 3.5 | - |
| MluI | 8260 | 6389 | 6383 | 5030 | 3323 | 29385 | 9.3 | CpG |
| MscI | 22014 | 17540 | 17473 | 14463 | 8487 | 79977 | 3.4 | Dcm |
| MseI | 317485 | 250113 | 253140 | 206683 | 115779 | 1143200 | 0.2 | - |
| NaeI | 36124 | 28390 | 29092 | 23567 | 13907 | 131080 | 2.1 | CpG |
| NarI | 24548 | 19480 | 19128 | 15350 | 9453 | 87959 | 3.1 | CpG |
| NcoI | 28322 | 22281 | 22396 | 18812 | 10777 | 102588 | 2.7 | - |
| NdeI | 23783 | 18259 | 18631 | 15259 | 8412 | 84344 | 3.2 | - |
| NgoMIV | 36124 | 28390 | 29092 | 23567 | 13907 | 131080 | 2.1 | CpG |
| NheI | 20817 | 16260 | 16969 | 13238 | 7689 | 74973 | 3.6 | CpG |
| NlaIII | 418130 | 330192 | 334785 | 273289 | 156669 | 1513065 | 0.2 | - |
| NruI | 6860 | 5757 | 5700 | 4567 | 2892 | 25776 | 10.6 | Dam, CpG |
| NsiI | 43428 | 34018 | 35033 | 28375 | 15713 | 156567 | 1.7 | - |
| PabI | 227668 | 178613 | 179848 | 143356 | 83731 | 813216 | 0.3 | ? |
| PciI | 33161 | 26046 | 26220 | 21355 | 11609 | 118391 | 2.3 | - |
| PmlI | 14886 | 11855 | 11831 | 9739 | 5735 | 54046 | 5.0 | CpG |
| PsiI | 21613 | 16489 | 17189 | 14630 | 8022 | 77943 | 3.5 | - |
| PspOMI | 12417 | 10033 | 9832 | 8367 | 5155 | 45804 | 5.9 | Dcm, CpG |
| PstI | 28575 | 22681 | 22851 | 18433 | 10705 | 103245 | 2.6 | - |
| PvuI | 14484 | 11857 | 11425 | 8577 | 5479 | 51822 | 5.2 | CpG |
| PvuII | 23329 | 18267 | 18288 | 14899 | 8578 | 83361 | 3.3 | - |
| RsaI | 227668 | 178613 | 179848 | 143356 | 83731 | 813216 | 0.3 | CpG |
| SacI | 23441 | 18743 | 19179 | 15464 | 9113 | 85940 | 3.2 | - |
| SacII | 17162 | 14260 | 13475 | 10456 | 7124 | 62477 | 4.4 | CpG |
| SalI | 13896 | 11211 | 11165 | 9109 | 5592 | 50973 | 5.3 | CpG |
| ScaI | 23150 | 18310 | 18575 | 14547 | 8612 | 83194 | 3.3 | - |
| SciI | 15474 | 12592 | 12683 | 10139 | 6318 | 57206 | 4.8 | ? |
| SelI | 199205 | 163018 | 159095 | 124218 | 81661 | 727197 | 0.4 | ? |
| SfoI | 24548 | 19480 | 19128 | 15350 | 9453 | 87959 | 3.1 | Dcm, CpG |
| SmaI | 15970 | 13459 | 13138 | 10592 | 7360 | 60519 | 4.5 | CpG |
| SnaBI | 11142 | 8720 | 8652 | 6557 | 3895 | 38966 | 7.0 | CpG |
| SpeI | 16496 | 13315 | 13289 | 10610 | 6130 | 59840 | 4.5 | - |
| SphI | 30758 | 24423 | 24743 | 19449 | 11603 | 110976 | 2.5 | - |
| SspI | 38664 | 30747 | 30468 | 25109 | 13899 | 138887 | 2.0 | - |
| StuI | 13261 | 10631 | 10759 | 8708 | 5063 | 48422 | 5.6 | Dcm |
| TaiI | 165258 | 131858 | 131619 | 104047 | 63412 | 596194 | 0.5 | CpG |
| TaqI | 230631 | 184035 | 185924 | 148946 | 90549 | 840085 | 0.3 | Dam |
| XbaI | 18663 | 14405 | 14392 | 11824 | 6540 | 65824 | 4.1 | Dam |
| XhoI | 15474 | 12592 | 12683 | 10139 | 6318 | 57206 | 4.8 | CpG |
| XmaI | 15970 | 13459 | 13138 | 10592 | 7360 | 60519 | 4.5 | CpG |
| ZraI | 11062 | 8886 | 8942 | 7191 | 4443 | 40524 | 6.7 | CpG |

? information not available
